# Dealing with ambiguity: Israeli physician’s attitudes and practices regarding pre-exercise certificates: a questionnaire study

**DOI:** 10.1186/s13584-016-0066-7

**Published:** 2016-05-01

**Authors:** Robert D Hoffman, Ron Golan, Shlomo Vinker

**Affiliations:** Maccabi Health Services, Gordon Street 8, Rehovot, Israel; Sports Medicine Department, Meuhedet Health Services, Tel Aviv, Israel; Leumit Health Services, Tel Aviv, Israel; Department of Family Medicine, Tel Aviv University, Tel Aviv, Israel

**Keywords:** Guidelines, Health legislation, Physician-patient relations, Preventive Medicine, Health promotion, Sports medicine, Physical fitness

## Abstract

**Background:**

It has become clear in recent years that a healthy lifestyle, including physical exercise is crucial for health maintenance. Nevertheless, most people do not exercise regularly. Physician intervention is beneficial in increasing patient exercise. In Israel, the 1994 “Sports Law” regarding exercising in a gymnasium requires a physician’s written authorization, but does not direct the physicians what they should ascertain before issuing the certificate. This pre-exercise certificate has been widely discussed in Israel over the last year as the law is to be revised to enable using a modification of the PAR-Q+ (Physical Activity Readiness questionnaire) patient questionnaire as a screening tool. This will leave the requirement for a pre-exercise certificate for a less healthy population, yet without clear instructions to the primary care physician on criteria for ascertaining fitness. Our aim was to evaluate how primary care physicians deal with the ambiguity of defining health criteria for issuing exercise authorization/certificate.

**Methods:**

We used an anonymous ten-item attitude/knowledge multiple choice questionnaire with an additional 13 personal/education and employment questions. We assessed each potential predictor of physician attitude and knowledge in univariate models.

**Results:**

135 useable questionnaires were collected. Of these, 43.7 % of the doctors will provide the pre-exercise certificate to all their patients; 63 % were aware of their HMO/employers guidelines for issuing certificates; 62 % stated they complied with these guidelines, and 16 % stated they did not follow them. In addition, 70 % of the physicians reported regular exercise themselves, an average of 4.12 h/week. These physicians tended to provide the pre-exercise certificate to all patients unconditionally, as compared to physicians that did not exercise regularly. (46 % vs. 14.5 %, *p* < 0.01)

**Conclusions:**

Most Israeli primary care physicians will provide the required certificate allowing their patients to exercise in the gym. There is a wide variation as to what physicians check before providing the certificate. The modification of the law has made the need for standardization of the nature of what is expected of primary care physicians more urgent. A large portion of physicians exercise on a regular basis – and exercising physicians are more positive regarding pre-exercise certificates.

Our study clearly shows a gap in knowledge transfer; and we call for a standardized approach to pre-exercise certificates utilizing computerized patient medical files.

## Background

The importance of physical exercise and the benefits of a healthy lifestyle are generally accepted [1–5]. The WHO set physical inactivity as the fourth leading risk factor for global mortality (6 % of deaths globally) [6]. Participation in 150 min of moderate physical activity a week is estimated to reduce the risk of ischemic heart disease by approximately 30 %, the risk of diabetes by 27 % and the risk of breast and colon cancer by 21–25 %. Nevertheless, levels of physical inactivity are rising in many countries with major implications for the general health of people worldwide [7]. Indeed, only approximately 30 % of Israelis engage in regular physical activity [8]. Most people in the Western World do not exercise regularly and only some of the population are reaching the cardiovascular goal of 2.5 h of weekly aerobic activity [7, 9]. WHO estimates in 2008 stated that 31 % of adults were insufficiently active (men 28 % and women 34 %) [6].

Physician intervention seems to be beneficial in increasing patient exercise patterns, and recommendations to increase physical exercise are a major component of all primary care lifestyle counseling [10, 11]. The 2012 review of 15 Randomized Controlled Trials of physical activity promotion has shown an outcome of significantly increased physical activity levels at 12 months following physician recommendation [12]. Conversely, another systematic review and meta-analysis assessed impact of exercise referral schemes on physical activity and health outcomes [13] and found only marginal benefits for sedentary patients. Nevertheless, while the significance of physician referral impact is still debated, the US Preventive Services Task Force has revised its 2002 recommendations to support behavioral counseling in primary care settings to promote physical activity [14].

The recent “Global Survey of Sports Medicine Doctors’ Attitudes and Practices in Recommending Physical Activity and Exercise to Patients” [15], an internet survey performed by the Institute of Lifestyle Medicine (ILM), examined physicians attitudes and referral patterns for exercise and physical activity. It found that 74 % of the respondents indicated that they *recommend* exercise. This study revealed that the likelihood of recommending exercise was associated with personal familiarity (i.e., physician engagement) with exercise and the availability of time in the patient visit.

However, a more complicated situation exists in Israel, in which the 1994 “Gymnasium Law” [16], requires each person who wants to use a gymnasium to bring a valid physician’s written authorization. Yet, there are no standard requirements on what basis such an authorization should be provided. The required pre-exercise certificate has been widely discussed in Israel over the last years. The law has been revised to allow using a modification of the PAR-Q+ patient questionnaire [17] as a screening tool. In the proposed legislation [18], exercise facilities are now allowed to accept a patient completed PAR-Q+ form in lieu of the physician certificate. The modification of the law has not changed the ambiguous nature of what is expected of primary care physicians, although it will appreciably reduce the volume of required physician certificates. Some of the HMO’s in Israel have directed their physicians to not provide pre-exercise certificates. Instead, the physicians are instructed to provide their patient with a general declaration of their overall medical status. This ambiguity causes a dilemma for primary care physicians, having to choose between employer directives and patients’ needs. As a result some patients have to consult special sports physicians in order to get the certificate and must pay privately (as it is not covered by the national health law coverage).

Indeed, the issue of willingness to provide pre-exercise certificates is controversial among family physicians; in terms of what efforts they will go to in order to satisfy the HMO, the patient and their own professional integrity. This creates a difficulty in procuring a pre-exercise certificate from HMO physicians, which prevents some people from regular exercising in a gym [8].

We therefore aimed to evaluate the current practice of Israeli physicians with regard to the above described dilemma. Furthermore, we analyzed whether personal lifestyle and exercise behavior of the physician had an effect on their willingness to provide a pre-exercise certificate to their patients.

## Methods

We used a “convenience sample” and distributed our questionnaires in CME programs for family physicians and trainees throughout Israel (in all academic departments of family medicine, covering all HMOs) and in a network of family physicians most of them active physicians, board certified and involved in teaching. Anonymous study questionnaires were disseminated in three different forums of Family Medicine physicians:

1. The internet forum “RAMBAM” (Israeli Research Network in Family Medicine), that has approximately 120 active members. This internet forum of researchers in Family Medicine encompasses many of the academic family physicians active in research and training. These physicians are the leaders of Israeli family medicine and include the heads of all academic Family Medicine training programs.
2. The survey was distributed to residents in family medicine training programs (approximately 50 residents of the 250 participating in the CME program), in order to sample a wide range of seniority.
3. The survey questionnaires were distributed at primary care conferences limited to single HMO’s in Israel in order to obtain samples from the different HMO’s.

These conferences typically host 50–100 primary care physicians. The physicians were asked to fill the questionnaire only once.

It is not possible to calculate the exact response rate. Our sampling consisted of a stand at conferences, and two internet mailings to the research network, we estimate that the response rate was between 25 and 45 % depending on the distribution method. When the questionnaire was distributed in small groups, response was higher, and when distributed via the internet, it was lower. The 85 specialists in Family Medicine who responded do represent approximately 5 % of the specialists in Israel.

Moreover, the responders include both leaders in the field as well as residents, and less senior family physicians.

The local IRB approved the study and waived the need for informed consent for this study format.

The questionnaire consisted of ten items about attitude and knowledge as well as 13 additional personal questions about medical education and employment, including hours of exercise per week. In the questionnaire, we examined the following issues:

- Willingness to provide pre-exercise certificates.
- Actions taken prior to providing pre-exercise certificates.
- Knowledge of employer’s recommendations.
- Personal participation in weekly exercise.

### Statistical analysis

All statistical analyses were performed using the Statistical Package for Social Sciences 22. We used descriptive statistics of physicians demographic, professional and exercise details and attitude/knowledge items. Each potential predictor of attitude/knowledge calculation was assessed in univariate models (*χ*^2^ for categorical variables and *t*-test for continuous variables). *P*-value < 0.05 was set to be significant.

## Results

Table 1 provides the demographic and professional background of the 135 participants. We found that 63 % of the respondents were aware of their HMO/employers guidelines for issuing pre-exercise certificates. 63 % stated they worked according to these guidelines, and 16 % stated they did not follow their HMO guidelines, while the others admitted to not being sure. Almost all doctors declared that they will provide the pre-exercise certificate (only one respondent said he would refuse). 43 % would provide it upon request without any further actions (Tables 2 and 3), but most doctors (78 %) will provide the pre-exercise certificate only after taking a pertinent history and writing the details in the patients file.

**Table 1:** Demographic and professional background of 135 participant physicians

	Number of Respondents	
Gender	130	56.9 % Female
Age	115	Range30-69 Average 45.4 (SD = 8.8)
Birthplace	127	53.5 % Israel
Year of MD graduation	126	1989.9 (SD = 8.9)
Country of MD training	129	62.8 % Israel 17.8 % former USSR 11.6 % Europe/America
Specialization	125	66.4 % Family Medicine (83) 16 % Residents (20) 17.6 % Other specialization or non-specialists (22)
Form of employment	125	80 % Employed (100) 20 % Self Employed (25)
Sports Medicine Specialization	128	4.7 % (6)
Weekly Hours of Exercise	132	Range 0–10 Average 2.8 (SD = 2.5)

**Table 2:** Providing a pre-exercise certificate to general population and cardiac patients. Certificate for the general population

	Affirmative response
Do you generally provide your patients with a pre-exercise certificate?	Yes – 43.7 %
	Maybe – 55.6 %
	No – 0.7 %
Do you Provide a certificate with no further action	18.5 %
Do you Refer to an exercise stress test (ergometry)	21.3 %
Do you Refer to a sports medicine consultant	8.1 %
Do you Refer to a cardiologist	11.0 %
Do you Ascertain that has recently undergone an ergometry exam	17.6 %
Do you Document the details of physical activity in the patient’s medical record	25.7 %
Do you Document negative symptoms in the patient medical record: (shortness of breath, chest pain/pressure or dizziness during exercise, loss of consciousness during or after exercise)	66.2 %
Do you Document pertinent family history in the patient’s medical record (sudden death at a young age; cardiovascular disease at a young age)	61.8 %

**Table 3:** Providing a pre-exercise certificate to general population and cardiac patients. Certificate for Cardiac patient

Negative Exercise stress test (ergometry) in the last year?	64.3 %
Negative Exercise stress test (ergometry) in the last two years?	31.6 %
Negative Exercise stress test (ergometry) in the last five years?	4.2 %
Do you provide Ischemic Cardiac patients (Angioplasty, CABG, positive stress test) with a pre-exercise certificate?	
Yes - 5.3 %	
Yes, under the limitations I explain – 18.0 %	
No, I refer to a sports physician – 12.0 %	
No, I refer to a cardiologist −53.4 %	
No, I refer to a cardiac rehabilitation program (exercise under medical supervision) – 11.3 %	

A large majority of physicians use a resting ECG and blood pressure measurement as part of their physical examination before issuing the pre-exercise certificate. Most doctors (77 %) would not provide a pre-exercise certificate to patients with a history of heart disease, and would refer these patients to a cardiologist, cardiac rehabilitation unit, or a sports physician.

Physicians that exercise were more likely to provide pre-exercise certificates. When analyzing the exercising habits of the physicians we surveyed, we found that 70 % of the participants exercise on a regular basis. Physicians who exercise demonstrate different behavior according to our survey. They are more aware of recommended guidelines (72 % vs. 52 %, *p* < 0.05), and tend to provide the pre-exercise certificate to all patients (46 % vs. 14.5 %, *p* = 0.005). Of the 43.7 % of physicians who would issue a pre-exercise certificate to all those requesting, most (81 %) exercise on a regular basis. A larger proportion of “exercising” physicians measure their patients BMI and anthropomorphic assessment (33 % vs. 10 %, *p* = 0.004).

There was a separate set of questions about the “cardiac patient” (Table 3). “Exercising” physicians are also more willing to certify cardiac patients for exercise (30 % vs. 12 %, *p* < 0.01). Moreover, these physicians send patients for stress tests more frequently than non-exercising physicians (25.3 % vs. 12.2 % *p* < 0.05).

Interestingly, whereas 73 % of Family Medicine specialists exercise on a regular basis, only 35 % of residents were exercising (*p* < 0.001). Table 4 presents the physical examination and ancillary tests prior to providing pre-exercise certificate.

**Table 4:** Physical examination and ancillary tests prior to providing Pre-exercise Certificate

Do you perform an examination with patients prior to providing Pre-exercise Certificate?	
Physical examination:	
Yes – to all patients	58.3 %
Depending on age/contents of patient file	29.9 %
No, Generally reading patient file sufficient (maybe check blood pressure)	11.8 %
Cardiovascular system: (Peripheral pulses, heart sounds and murmurs, including Carotid artery)	91.1 %
Musculoskeletal system: (Muscle strength, range of motion and joint stability)	20.0 %
Nervous system: (Reflexes, Neurological deficits, Romberg test)	7.4 %
Vision and Hearing	7.4 %
Glands (thyroid and Lymph nodes)	3.0 %
Abdominal: (Organomegaly – Liver, Spleen, Gall Bladder)	8.1 %
Resting ECG and Blood Pressure	74.1 %
Sub-maximal Ergonometric ECG Testing: (up to 85 % of maximal predicted pulse per age/sex)	10.4 %
Maximal Ergonometric ECG Testing: (up to 90 % of maximal predicted pulse per age/sex)	20.0 %
Blood tests	9.6 %
Lung function tests: (Peak Flow, Flow-Volume Loop)	4.4 %
Anthropomorphic measurements: (Weight, Height, BMI, Fat tissue percentage)	23.7 %

## Discussion

There is no doubt that increased physical fitness is an important goal for Primary Care Physicians and their patients, with a potential for a major impact on health [19, 20]. A study in 2002 demonstrated that the general relative risk of death for those in the lowest quintile of fitness was four times that of those in the highest quintile of fitness [21, 22]. Therefore, it is reasonable for primary care physicians to encourage physical activity of their patients.

Indeed, a recent study performed in Spain showed that if Primary Care Physicians actively encourage physical activity, a significant increase can be achieved [19]. However, recent systematic review and meta-analysis have found these interventions to have only marginal benefits for sedentary patients, after following them for a longer period of time [22].

Importantly, several unresolved question emerge from our survey: How should a reasonable physician screen patients before certifying exercise [23, 24]?

The use of a PAR-Q+ (physical activity readiness questionnaire) as recommended in the recent legislation will screen out many of the patients. If there are no positive responses in the PAR-Q survey, the patient is to be qualified without further testing. If there is a positive response, − a further workup is mandated. These recommendations are in accordance with Scheinowitz’s 2008 suggestions for “Pre-participation screening of individuals engaging in noncompetitive physical activity” [8] as well as the Canadian PAR-Q+ collaboration [17].

Our survey showed very common use of a resting ECG as part of the pre-exercise certificate exam, even though this was not part of the suggested 2008 guidelines. Interestingly, this suggested recommendation was not supported in a 2011 study regarding the reduction in risk of sudden death among Israeli athletes [25].

These results emphasize the need for clear, unified guidelines as to what is required in order to provide a pre-exercise certificate.

Our results show that virtually all physicians will provide a pre-exercise certificate for at least some of their patients. Most physicians used the basics – a medical history and physical exam, documented in the patient file, before providing the pre-exercise certificate. Many of those surveyed (27.7 %) felt a stress ECG necessary for a pre-exercise certificate.

Another unresolved issue emerging from our study is the lack of knowledge regarding current guidelines for providing pre-exercise certificates.

Most physicians were unaware of their detailed employers guidelines regarding pre-exercise certificates, and many admitted to not abiding by these guidelines.

This is to be expected in the ambiguous situation we have described, in which patients are obliged to obtain a pre-exercise certificate, but there are no clear requirements or Israeli guidelines dictating the terms for providing this certificate. Most doctors (78 %) will provide a pre-exercise certificate only after taking a pertinent history and writing the details in the patients file, suggesting that many physicians are willing to facilitate their patients exercise, and are less concerned with guidelines and may be unaware of the medico-legal issues.

We found clear differences between physicians that exercise regularly and those who don’t exercise, in regards to their attitudes towards pre-exercise certificates. Our finding that there are significant differences in reported pre-exercise certificate exams between “exercising” physicians and non-exercising physicians, suggests that by encouraging physician exercise [26], we may have an effect on the physicians’ patients too.

## Policy implications

We have shown that under the current law requiring patients to obtain a pre-exercise certificate there is a large variation in physicians’ performance. This is related, at least in part, to the lack of specific guidelines indicating the format of the certificate and the evidence-based criteria that should be used in issuing the certificate. Furthermore, it is complicated by the fact that some HMOs have advised their physicians not to issue such certificates and asked for a Sports Medicine specialist authorization (which in turn is a service that is not reimbursed). With the recent changes of the law there is a new opportunity to improve the health of the Israeli population by ensuring that physicians counsel their patients about the importance of exercise and use standard criteria for issuing the required pre-exercise certificates. It also is essential that the HMOs comply with the law. We have suggested policies that should be discussed and ideally adopted by the Ministry of Health that would achieve those goals.

Our findings are consistent with the urgent need for new Israeli regulations and guidelines in the pre-exercise certificates. These guidelines should be written and agreed upon by all the relevant disciplines (Sports medicine, Family Medicine, Cardiology, Orthopedic surgeons etc.) From our point of view, it has become an urgent topic in the light of the new legislation. The new situation, where all patients requesting a pre-exercise certificate from their primary care physician have “failed” at least one question in the PAR-Q+ will make the family physicians even more ambiguous about the topic. We fear that the scale of over-testing and improper testing before giving the certificate will increase substantially. Israel’s primary health care system with its universal computerized medical files offers an opportunity for a solution. If the HMOs or Ministry of Health can adopt a national guideline for requirements necessary for providing pre-exercise certificates, these could readily be incorporated into the HMO’s medical records software. This would enable all physicians in the primary care system to provide pre-exercise certificates, in accordance with the preset suggested guideline requirements.

## Methodology limitations

The majority of those surveyed are board certified in Family Medicine, as are the majority of specialists in Israeli primary care. Our sample is representative in gender distribution. It over- represents young, Israeli born, board certificated family physicians because this is the group that tends to come to academic CME programs and participate in the online research discussion group. The comparative demographics can be seen in Table 5.

**Table 5:** Comparison of study demographics with Israeli primary care physicians

	Study demographics	Israeli primary care physicians demographics^a^
Gender	56.9 % Female	45 % Female (of primary care physicians)
Age	Range 30–69 Average 45.4 SD 8.8	36 % ages 25–44 62 % ages 45–64 (of all family physicians)
Country of Birth	53.5 % Israel	34.5 % Israel (of all physicians)
Year of MD graduation	Average 1989 SD 8.9	No data found
Country of MD training	62.8 % Israel 17.8 % former USSR 11.6 % Europe/America	35.7 % Israel
		41.1 % Former USSR
		24 % Europe/America (of all physicians)
Specialization	66.4 % Family Medicine 16 % Residents 17.6 % Other specialization or non-specialists	Family Medicine 57 % (of primary care specialists)
Form of employment	80 % Employed 20 % Self Employed	62 % Employed 6 % Self Employed 32 % Both
HMO	44.4 % Clalit 43 % Maccabi 11 % Meuhedet 2.4 % Leumit	52 % Clalit 25 % Maccabi 13.6 % Meuhedet 9 % Leumit (of all physicians)
Place of employment	80 % community clinic 14 % rural community clinic 3.2 % IDF military clinics 2.4 % mostly in hospitals	79 % community clinics (of Family Medicine specialists) _________ 39 % community clinics 54 % hospitals (of all physicians) _________ Population in urban localities: 91.2 % Population in rural localities: 8.8 % (2014)

^a^Israeli primary care physicians’ demographics obtained from the Israeli Ministry of Health 6.2015 report on “Medicine in the Community: physicians working in Family Medicine in Israel” [27]

The response rate is not optimal. We estimate that the response rate was between 25 and 45 % depending on the distribution method.

Our sample is more representative of the opinion leaders in Family Medicine and the future generation of residents on the cusp of finishing their training, who are now the young specialists. Our sample is less representative of the older generation, and is not representative of the non-specialists in primary care. As the main result of the study is the finding that there is not a standard criteria used by responding physicians before filling in the pre-exercise certificate, the sample methods used allowed us to gain important data regarding primary care physician behavior.

The survey was voluntary, and may be biased towards physicians with a greater interest in exercise.

## Conclusion

In conclusion, our results imply that the situation, in which physicians individually try to cope with an ambiguous law regarding gymnasium exercise’s authorization, must be resolved.

We do not recommend another change in the law, but rather call for explicit national guidelines to be provided to the primary care physicians being asked to sign the pre-exercise certificates.
